# Environmental Factors Develop Different Patterns of Immune Disease

**DOI:** 10.1289/ehp.1104043

**Published:** 2011-09-01

**Authors:** Luis M. Blasco

**Affiliations:** UARH, Hospital Marqués de Valdecilla, Santander, Spain, E-mail: grullus99@yahoo.es

I read with interest the article by Schmidt (2011) on the sprawling explosion of autoimmune diseases and its link to environmental exposure. Schmidt (2011) summarized the problematic state of the field: Systemic autoimmune diseases are common but thought rare; their clinical identification is far from the medical school description; and they continue to be identified as an autoantibody–target–manifestation scheme. Experience shows that a patient develops different autoantibodies through the lifespan, with different clinical patterns within each phase; deeper investigation shows that organ autoimmune disease is in fact systemic. Likewise, allergy, food intolerance, cancer, and immunodeficiency (all broad diseases that are immune in nature) cross and share autoimmunity. This suggests that immature immune systems are promoted and prevented from natural selection in the era of antibiotics, but they pay the cost of fostered health dysfunctions or diseases exposed to the current complex hostile environment.

I noticed this complex scenario in a survey of 22 patients reporting sick building syndrome (Blasco 2011). Although reported data was limited to autoimmune cases and the involved substances were not yet identified, I found that the same environment triggered and worsened other immune disorders. The health of two patients with asthma inexplicably worsened when they started to work in the building. One patient developed gynecological cancer; another patient, who had a past history of Hodgkin’s lymphoma, developed chronic fever and fatigue again that lasted 3 years, until she was relocated.  Some of the patients reported new adult onset of clinical intolerance of milk or other foods, and one patient was positive in a breath test for lactose intolerance. A review of family histories revealed that in 20% of the patients, more than one direct relative was affected by cancer. Personnel records showed that allergy was present in 59% of the patients; recurrent infections during childhood were common; 20% required amigdalectomy. One patient suffered rheumatic fever; one patient had not been effectively immunized after repeated hepatitis vaccines; and another had defective CD4 and suffered recurrent pneumococcal infections.

It would be surprising if these illnesses did not share a common root in the immune system. Schmidt (2011) underlined rising prevalence rates of autoimmunity and discussed causes. I believe that this trend is relevant in general to immune disorders because of different reactions within the same scope of lymphocyte dysfunction in response to our new aggressive environment.
